# CRISPR-Cas9-mediated IncF plasmid curing in extraintestinal pathogenic *Escherichia coli*

**DOI:** 10.1128/spectrum.03692-23

**Published:** 2023-11-29

**Authors:** Liang Chen, Gisele Peirano, Kelly Yen, Bingjie Wang, Austin Terlecky, Rebekah DeVinney, Barry N. Kreiswirth, Johann D. D. Pitout

**Affiliations:** 1 Center for Discovery and Innovation, Hackensack-Meridian Health, Nutley, New Jersey, USA; 2 Hackensack Meridian School of Medicine, Nutley, New Jersey, USA; 3 Cummings School of Medicine, University of Calgary, Calgary, Alberta, Canada; 4 Alberta Precision Laboratories, Calgary, Alberta, Canada; 5 Department of Clinical Laboratory Medicine, Shanghai Pulmonary Hospital, Tongji University School of Medicine, Shanghai, China; 6 University of Pretoria, Pretoria, Gauteng, South Africa; JMI Laboratories, North Liberty, Iowa, USA

**Keywords:** extraintestinal pathogenic *Escherichia coli*, plasmid, CRISPR, pCasCure

## Abstract

IncF plasmids are commonly found in extra-intestinal pathogenic *Escherichia coli* (ExPEC) strains, serving as reservoirs for antimicrobial resistance (AMR) genes and virulence factors, persistently coexisting with ExPEC lineages. Multidrug-resistant (MDR) high-risk ExPEC clones, particularly ST131, ST1193, and ST410, have acquired diverse IncF plasmids over time, containing various AMR determinants, contributing significantly to their global success. However, the broader roles of these IncF plasmids in the success of MDR ExPEC clones, beyond AMR, remain elusive. In this study, we employed a novel clustered regularly interspaced short palindromic repeats–CRISPR-associated protein-9 nuclease (CRISPR-Cas9)-mediated pCasCure plasmid-curing system to precisely remove specific IncF plasmids among ExPEC clones (ST1193, ST131, and ST410). Antibiotic-resistant parent strains reverted to antibiotic-susceptible states post-curing; however, IncF plasmid curing did not show significant impact on bacterial *in vitro* growth and had little impact on other *in vitro* phenotypes, including survival in water, dry environment and biofilm production. In addition, IncF plasmid curing did not affect the conjugation frequency of KPC-producing pKpQIL plasmid. This study represents a pivotal initial step in understanding the precise roles of IncF plasmids in the success of ExPEC. Future research will be crucial in investigating their influence on cell invasion and *in vivo* fitness, thereby providing a more comprehensive perspective on the functions of IncF plasmids in MDR ExPEC clones.

**IMPORTANCE:**

Understanding the role of IncF plasmids in the success of drug-resistant bacteria has far-reaching implications for tackling antibiotic resistance. The study's use of a novel CRISPR-Cas9-mediated plasmid-curing system provides a precision tool for dissecting the specific impact of IncF plasmids on ExPEC clones, especially high-risk, multidrug-resistant strains like ST131, ST1193, and ST410. The study offers a crucial stepping stone for future research into understanding how these plasmids influence more complex aspects of bacterial behavior, such as cell invasion and *in vivo* fitness.

## OBSERVATION

IncFs are the most abundant plasmid types among extra-intestinal pathogenic *Escherichia coli* (ExPEC) (1). IncF plasmids are large (>50 kb), mosaic, and contain antimicrobial resistance (AMR) genes, virulence associated factor genes, various addiction, restriction, and truncated transfer systems (2). The presence of addition/restriction systems, combined with truncated transfer regions, has led to IncF plasmid persistence with subsequent fixation within certain ExPEC lineages (3).

Certain ExPEC clones, such as ST131, ST1193, and ST410, are dominant among multidrug resistant (MDR) ExPEC populations (4 – 6). These MDR high-risk clones acquired different types of IncF plasmids over time that contain various AMR determinants including CTX-M and carbapenemase genes (3, 7). IncF plasmids have been pivotal in the global success of pandemic MDR high-risk clones such as ST1193, ST131, and ST410 (3).

The clustered regularly interspaced short palindromic repeats–CRISPR-associated protein-9 nuclease (CRISPR-Cas9) system is a novel tool to generate site-speciﬁc double-strand breaks for genome editing in mammalian cells, plants, fungi, and bacteria (8, 9). We previously developed a novel CRISPR-Cas9-mediated pCasCure plasmid-curing system that can precisely cure specific antimicrobial genes and plasmids in bacterial hosts (10). Currently, limited information is available on which specific biological features on IncF plasmids have contributed to the success of MDR ExPEC clones (3, 7, 11). In this study, we employed the pCasCure system to remove specific IncF plasmids among MDR high-risk ExPEC clones belonging to ST1193, ST131, and ST410, to assess the impact of IncF plasmids on ExPEC antimicrobial resistance and *in vitro* phenotypes. Our study represents a first step in utilizing CRISPR-Cas9-mediated plasmid curing system toward deciphering the roles of IncF plasmids that contribute to the success of ExPEC lineages.

**Fig 1:**
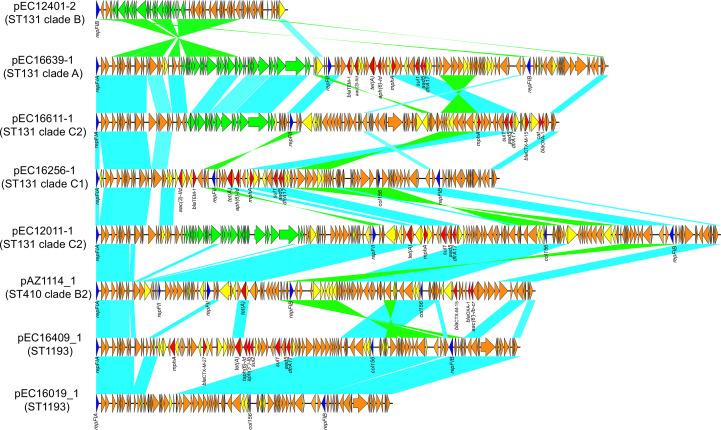
Comparative analysis of IncF plasmids cured in ExPEC strains. Light blue shading represents shared regions of homology, while light green shading indicates reversed displayed homology. Open reading frames (ORFs) are depicted as arrows and colored according to predicted gene function. Orange arrows represent plasmid scaffold regions. Genes associated with the *tra* locus are indicated by green arrows, and replication-associated genes are denoted by dark blue arrows. Antimicrobial resistance genes are highlighted with red arrows, while accessory genes are marked with yellow arrows.

Eight clinical *Escherichia coli* strains, which were previously responsible for bloodstream and urinary tract infections (12 – 14), were included in this study (Table 1; Table S1): EC16639 (ST131 clade A), EC12401 (ST131 clade B), EC16256 (ST131 subclade C1), EC12011 (ST131 subclade C2), EC16611 (ST131 subclade C2), EC16019 (ST1193), EC16409 (ST1193), and AZ1114 (ST410 subclade B2). All strains underwent long- and short-read sequencing using Oxford Nanopore and Illumina HiSeq platforms. Hybrid assembly using both Nanopore and Illumina reads (15) led to the complete closure of the eight strains. Sequence analysis showed that EC16639 (ST131-A), EC16256 (ST131-C1), and EC12011 (ST131-C2) each contained a single plasmid; EC12401 (ST131-B) and EC16019 (ST1193) contained three plasmids; EC16611 (ST131-C2), EC16409 (ST1193), and AZ1114 (ST410 subclade B2) each contained five plasmids (Table S1). The following IncF plasmids (*n* = 8) ranging from 51 to 167 kb, were selected for curing: (Table 1; Fig. 1) pEC16639_1 (F1:A2:B20), pEC12401_2 (F-:A-:B10), pEC16256_1 (F1:A2:B20), pEC12011_1 (F36:A4:B1), pEC16611_1 (F2:A1:B-), pEC16019_1 (F-:A1:B10), pEC16409_1 (F-:A1:B10), and pAZ1114_1 (F36:A4:B1). The different AMRs present on each plasmid are listed in Table 1; Table S1; Fig. 1. The *bla*_CTX-M-14_ and *bla*_CTX-M-15_ were integrated in the chromosomes of EC16256 (ST131-C1) and EC12011 (ST131-C2), respectively.

**TABLE 1:** *Escherichia coli* strains, IncF plasmid structures, curing, antimicrobial resistance genes, and antimicrobial susceptibilities^*a*^ ^,^ ^*b*^

Strain (no. of plasmids)	ST	IncF plasmids (size kb) pMLST	Cured plasmid (size kb) AMR genes	AMP	AMC	CZ	CRO	CAZ	FEP	MEM	CIP	GEN	TOB	AMK	TET	SXT
EC16639 (*n* = 1)	131-A	pEC16639_1 (137) F1:A2:B20	pEC16639_1 (137) *bla*_TEM-1,_ *aac(3)-IId*, *tet(A)*, *aph(6)-Id*, *aph(3’)-Ib*, *mphA, sul1*, *sul2*, *aadA5*, *dfrA17*, *strAB*, *qacEdelta1*	≥32	2/1	16	0.25	0.25	0.12	<0.5	≥8	≥32	≥32	0.5	8	>4/76
EC16639-C (*n* = 0)	131-A	–^*c*^	–	2	2/1	4	0.25	0.25	0.12	<0.5	≥8	1	1	0.5	1	0.5/9.5
EC12401 (*n* = 3)	131-B	pEC12401_2 (51) F-:A-:B10 pEC12401_1 (65) F2:A-:B10	pEC12401_2 (51) No AMR genes	2	2/1	2	0.25	0.25	0.12	<0.5	0.12	1	1	0.5	1	0.5/9.5
EC12401-C (*n* = 2)	131-B	pEC12401_1 (65) F2:A-:B10	–	2	2/1	2	0.25	0.25	0.12	<0.5	0.12	1	1	0.5	1	0.5/9.5
EC16256 (*n* = 1)	131-C1	pEC16256_1 (108) F1:A2:B20	pEC16256_1 (108) *aac(3)-IId;aadA5;aph(3'')-Ib;aph(6)-Id;bla*_TEM-1_ *;qacEdelta1;sul1;sul2;tet(A);dfrA17;mphA*	≥32	4/2	≥32	≥32	4	≥32	<0.5	≥8	≥32	≥32	0.5	16	>4/76
EC16256-C (*n* = 0)	131-C1	–	–	≥32	4/2	≥32	≥32	4	≥32	<0.5	≥8	1	1	0.5	2	0.5/9.5
EC12011 (*n* = 1)	131-C2	pEC12011_1 (167) F36:A4:B1	pEC12011_1 (167) *aadA5;qacEdelta1;sul1;tet(A);dfrA17*,*mphA*	≥32	4/2	≥32	≥32	≥32	≥32	<0.5	≥8	4	2	0.25	16	>4/76
EC12011-C (*n* = 0)	131-C2	–	–	≥32	4/2	≥32	≥32	≥32	≥32	<0.5	≥8	2	2	0.25	1	1/19
EC16611 (*n* = 5)	131-C2	pEC16611_1 (124) F2:A1:B-	pEC16611_1 (124) *aadA5;bla*_OXA-1_ *;bla*_CTX-M-15_ *;catB3;qacEdelta1;sul1;dfrA17*	≥32	32/16	≥32	≥32	16	≥32	<0.5	≥8	4	2	0.25	1	>4/76
EC16611-C (*n* = 4)	131-C2	–	–	8	8/4	16	0.25	0.25	0.12	<0.5	≥8	2	2	0.25	1	1/19
EC16019 (*n* = 3)	1193	pEC16019_1 (79) F-:A1:B10	pEC16019_1 (79) No AMR genes	≥32	4/2	≥32	≥32	16	≥32	<0.5	≥8	≥32	≥32	0.5	1	1/19
EC16019-C (*n* = 2)	1193	–	–	≥32	4/2	≥32	≥32	16	≥32	<0.5	≥8	≥32	≥32	0.5	1	1/19
EC16409 (*n* = 5)	1193	pEC16409_1 (114) F-:A1:B10	pEC16409_1 (114) *aadA5;aph(3'')-Ib;aph(6)-Id;bla*_CTX-M-27_ *;qacEdelta1;sul1;sul2;tet(A);dfrA17;mphA;strAB*	≥32	8/4	≥32	≥32	8	≥32	<0.5	≥8	4	1	0.25	16	>4/76
EC16409-C (*n* = 4)	1193	–	–	4	4/2	4	0.12	0.25	0.12	<0.5	≥8	2	1	0.25	1	1/19
AZ1114 (*n* = 5)	410-B2	pAZ1114_1 (117) F36:A4:B1	pAZ1114_1 (117) *aac(6')-Ib-cr5;bla*_OXA-1_ *;bla*_CTX-M-15_ *;catB3;tet(A*)	≥32	≥32/16	≥32	≥32	≥32	≥32	8	≥8	4	≥32	32	16	1/19
AZ1114-C (*n* = 4)	410-B2	–	–	≥32	≥32/16	≥32	≥32	≥32	8	8	≥8	1	≥32	2	2	1/19

^*a*^ Sequence type (ST), antimicrobial resistance (AMR), ampicillin (AMP), amoxicillin-clavulanic acid (AMC), cefazolin (CF), ceftriaxone (CRO), ceftazidime (CAZ), cefepime (FEP), MEM (meropenem), ciprofloxacin (CIP), gentamicin (GEN), tobramycin (TOB), amikacin (AMK), tetracycline (TET), and trimethoprim-sulfamethoxazole (SXT).

^*b*^ EC16639-C (cured strain), EC12401-C (cured strain), EC16256-C (cured strain), EC12011-C (cured strain), EC16611-C (cured strain), EC16019-C (cured strain), EC16409-C (cured strain), and AZ1114 (cured strain).

^*c*^ "-" indicates negative for the genes or plasmids.

pCasCure plasmids containing IncF plasmid gRNA were constructed as previously described with minor modifications (10). In brief, we designed two 20-bp spacer sequence

(N20) targeting IncF plasmid replication genes, repFIB (AGTAGGCTGACTGTACCAGA) and repFIA (GGCGGCATATAGTCTCTCCC), using Geneious Prime. These N20 sequences were integrated into the *BsaI* sites in pCasCure-Apr following the method described previously (16). Subsequently, we electroporated the pCasCure-Apr plasmid with repFIB or repFIA gRNA into clinical *E. coli* isolates, followed by arabinose (1%) induction to activate Cas9 nuclease production. Confirmation of successful IncF plasmid curing was achieved through PCR targeting repFIB or repFIA. Selected plasmid-curing mutants underwent whole-genomic sequencing (WGS) using the Illumina HiSeq sequencer. WGS analysis confirmed the absence of the target IncF plasmids without any additional off-target mutations.

We initially assessed the impact of IncF plasmid curing on antimicrobial susceptibility. Custom-made microdilution panels were used for susceptibility testing to determine minimum inhibitory concentrations (MICs) to antibiotics following the Clinical Laboratory and Standards Institute guidelines (17) (Table 1). The MICs of the drugs correlated with the presence and absence of different plasmid AMR genes in the parental and their cured strains (Table 1). As an example: curing of pEC16639_1 [that contained *bla*_TEM-1_, *strAB*, *aac(3)-IId*, *tet(A)*, *aph(6)-Id*, *aph(3*′*)-Ib*, *mphA*, *sul1*, *sul2*, *aadA5*, and *dfrA17*] in EC16639 restored the susceptibilities to ampicillin, cefazolin, gentamicin, tobramycin, tetracycline, and trimethoprim-sulfamethoxazole (EC16639-C) (Table 1).

We then assessed *in vitro* phenotypes following plasmid curing, including growth, survival in water and in a dry environment, and biofilm production. Standard growth curves were generated using various media: Luria-Bertani, M9 minimal medium, artificial urine medium, and Davis-Mingoli medium, employing previously described methods (18). Briefly, overnight cultures were diluted 1:1,000 in the respective media. Subsequently, 150-µL aliquots were dispensed into flat-bottomed 96-well plates. Plates were sealed with breathable membranes and incubated at 37°C. Optical density (OD) at 600 nm was recorded at 10-minute intervals over 24 hours. Survival in water and dry conditions was evaluated with a method described previously, with minor modifications (18). Briefly, overnight cultures were diluted to approximately 10^8^ CFU and then inoculated into 10 mL of autoclaved tap water. Bacterial survival was assessed after 24 and 72 hours of incubation at 37°C through colony plating. For assessing survival in a dry environment, a 96-well flat-bottomed microtiter plate was utilized. Dry bacterial samples were exposed for 2, 5, and 7 hours at room temperature, followed by colony plating. Biofilm formation was evaluated using a 96-well polyvinyl chloride microtiter plate with a 24-hour incubation at 30°C using the crystal violet method (18). All the aforementioned *in vitro* assays were performed in at least three biological replicates, and each experiment was repeated twice.

Growth curve analysis revealed that plasmid curing had no discernible impact on the growth of cured strains in all eight pairs tested, which included Luria-Bertani, M9 minimal medium, artificial urine medium, and Davis-Mingoli medium (data not shown). This suggests that the IncF plasmids had a very limited effect on the growth fitness of the bacterial hosts among these epidemic MDR ExPEC strains. In terms of survival in water, all eight wild-type and IncF plasmid-cured pairs exhibited robust survival rates at 24, 48, and 72 hours, with growth increasing to over 250%–350% at 72 hours. However, no significant difference was observed between the wild-type and plasmid-cured pairs in terms of water survival (data not shown). Similarly, survival in a dry environment showed no significant differences between individual wild-type and plasmid-cured pairs. However, there were some strain-to-strain variations (Fig. S1). For example, ST1193 strain EC16019 displayed approximately 25% survival at 2 hours on a dry plate, while ST131-A strain EC16639, ST131-B strain EC12401, and ST410 strain AZ1114 showed less than 2% growth at 2 hours. Nevertheless, all strains, including both the wild-type and plasmid-cured mutants, exhibited low survival rates (< 3%) after 7 hours of exposure to a dry environment, suggesting that these MDR ExPEC strains do not survive well in environments lacking moisture. Regarding biofilm production, all eight pairs displayed low-level biofilm production [average optical density (OD) = 0.16] compared to the positive control strain, *Staphylococcus. epidermidis* strain RP62A (OD = 1.96) (Fig. S2). It is noted that IncF plasmid curing in ST131 C2 strain EC16611 and ST410 strain AZ1114 resulted in a significant reduction in biofilm formation compared with their wild-type strain. This suggests that plasmid-borne genes may play a role in the variation of biofilm production among clinical MDR ExPEC isolates.

Lastly, we investigated whether the presence of IncF plasmids had an impact on the acquisition of carbapenemase-producing plasmids. To address this, we conducted plasmid filter mating experiments using the epidemic IncFIIK2 *bla*_KPC_-harboring pKpQIL plasmid (pKpQIL-03) (19) as the vector. Diaminopimelic acid auxotrophic *E. coli* DH10B harboring pKpQIL-03 was used as the donor, transferring the plasmid into various *E. coli* pairs, except for AZ1114, which already contained a carbapenemase gene *bla*_VIM-1_ on an IncN plasmid. Transconjugants were selected on meropenem (2 µg/mL) plates and confirmed through PCR. The conjugation frequency was calculated as the ratio of the number of transconjugants to the number of recipients. The results revealed that the pKpQIL-03 plasmid successfully transferred into these ExPEC pairs, with a high conjugation frequency ranging from 10^−3^ to 10^−2^ (data not shown). However, there were no significant conjugation frequency differences observed between the wild-type and IncF plasmid-cured mutants. The results suggest that, despite both belonging to the same category of IncF plasmids, the IncF plasmids from clinical MDR ExPEC strains did not impede the acquisition of IncFIIK pKpQIL plasmids. This implies that IncF plasmids in MDR ExPEC strains might be compatible with IncFIIK pKpQIL plasmids.

In summary, this study represents one of the first investigations utilizing a precise CRISPR-Cas plasmid curing platform to specifically probe the role of IncF plasmids in MDR ExPEC strains. Our results clearly demonstrate that the successful removal of resistance plasmids can sensitize the bacteria to antibiotics once more. Considering the strong association between IncF plasmids and MDR ExPEC strains, we found that it is imperative to delve deeper into the functions of IncF plasmids beyond their involvement in antimicrobial resistance. One noteworthy discovery is that the IncF plasmid has a limited impact on the *in vitro* fitness of ExPEC hosts. This suggests that these plasmids may have well adapted with these bacterial hosts. To gain a more comprehensive understanding of the IncF plasmid’s role, further research is necessary to investigate its influence on cell invasion and *in vivo* fitness. This approach will provide an accurate characterization of how IncF plasmids contribute to the success of epidemic MDR ExPEC clones. Such data will aid in developing novel treatment strategies and provide novel genomic surveillance targets to track emerging MDR ExPEC clones. Future studies will use the same curing strategies to investigate the role of IncF plasmids among other ExPEC MDR clones (ST38, ST167, ST405, and ST648) and non-MDR high-risk ExPEC clones (ST69, ST73, and ST95).

## Data Availability

Sequences have been deposited at GenBank under BioProject accession number PRJNA549322.

## SUPPLEMENTAL MATERIAL

Supplemental material spectrum.03692-23-s0001.pdf Table S1; Figures S1 and S2.

ASM does not own the copyrights to Supplemental Material that may be linked to, or accessed through, an article. The authors have granted ASM a non-exclusive, world-wide license to publish the Supplemental Material files. Please contact the corresponding author directly for reuse.
