# Supplementary material for: CRISPR-Cas9-mediated IncF plasmid curing in extraintestinal pathogenic Escherichia coli
Source: Microbiol Spectr. 2023 Nov 29;12(1):e03692-23. doi: 10.1128/spectrum.03692-23 (PMC10783119; doi:10.1128/spectrum.03692-23)
Supplement: Supplemental material — Table S1; Figures S1 and S2. [file spectrum.03692-23-s0001.pdf]

Table S1. Genomic informaiton of ExPEC isolates

Strains	Chr/plasmid	Length (bp)	Plasmdi replicons	AMR genes	QRDR
AZ1114	AZ1114_chr	4729701	-		gyrA_D87N;gyrA_S83L;parC_S80I
	pAZ1114_1	117395	Col156;IncFIA;IncFIB	aac(6')-Ib-cr5;blaOXA-1;blaCTX-M-15;catB3;tet(A)	
	pAZ1114_2	90793	IncI1-I(Alpha)		
	pAZ1114_3	43860	IncN3	aac(6')-II;aac(6')-Ib-cr5;blaVIM-23;blaOXA-1;catB3;qacEdelta1;sul1	
	pAZ1114_4	4072	unknown		
	pAZ1114_5	3223	unknown		
EC12011	EC12011_chr	5242119	-	blaCTX-M-15	gyrA_D87N;gyrA_S83L;parC_E84V;parC_S80I;parE_I529L
	pEC12011_1	167222	Col156;IncFIA;IncFIB	aadA5;qacEdelta1;sul1;tet(A);dfrA17,mphA	
EC12401	EC12401_chr	5117098	-	aac(3)-IId;aadA2;blaTEM-1;qacEdelta1sul1;dfrA12	gyrA_S83L;parC_S80R;parE_I529L
	pEC12401_1	65097	IncFII		
	pEC12401_2	51361	IncFIB		
	pEC12401_3	45247	new		
EC16019	EC16019_chr	4988211	-	aac(3)-IIe;aac(6')-Ib-cr;blaCTX-M-15;blaOXA-1;catB3	gyrA_D87N;gyrA_S83L;parC_S80I;parE_L416F
	pEC16019_1	79420	Col156;IncFIA;IncFIB		
	pEC16019_2	7939	ColRNAI		
	pEC16019_3	2113	Col(BS512)		
EC16256	EC16256_chr	5090635	-	blaCTX-M-14	gyrA_D87N;gyrA_S83L;parC_E84V;parC_S80I;parE_I529L
	pEC16256_1	108010	Col156;IncFIA;IncFIB;IncFII	aac(3)-IId;aadA5;aph(3'')-Ib;aph(6)-Id;blaTEM-1;qacEdelta1;sul1;sul2;tet(A);dfrA17;mphA	
EC16409	EC16409_chr	5027666	-		gyrA_D87N;gyrA_S83L;parC_S80I;parE_L416F
	pEC16409_1	113567	Col156;IncFIA;IncFIB	aadA5;aph(3'')-Ib;aph(6)-Id;blaCTX-M-27;qacEdelta1;sul1;sul2;tet(A);dfrA17;mphA;strAB	
	pEC16409_2	60239	IncI2(Delta)		
	pEC16409_3	5165	Col156		
	pEC16409_4	3348	unknown		
	pEC16409_5	2113	unknown		
EC16611	EC16611_chr	5062930	-		gyrA_D87N;gyrA_S83L;parC_E84V;parC_S80I;parE_I529L
	pEC16611_1	123970	IncFIA;IncFII	aadA5;blaOXA-1;blaCTX-M-15;catB3;qacEdelta1;sul1;dfrA17	
	pEC16611_2	5164	Col156		
	pEC16611_3	5073	Col(pHAD28)		
	pEC16611_4	2149	Col(BS512)		
	pEC16611_5	1549	Col(MG828)		
EC16639	EC16639_chr	4878466	-		gyrA_D87N;gyrA_S83L;parC_S80I;parE_E460D
	pEC16639_1	137178	IncFIA;IncFIB;IncFII	aac(3)-IId;aadA5;aph(3'')-Ib;aph(6)-Id;blaTEM-1;qacEdelta1;sul1;sul2;tet(A);dfrA17;strAB;mphA	

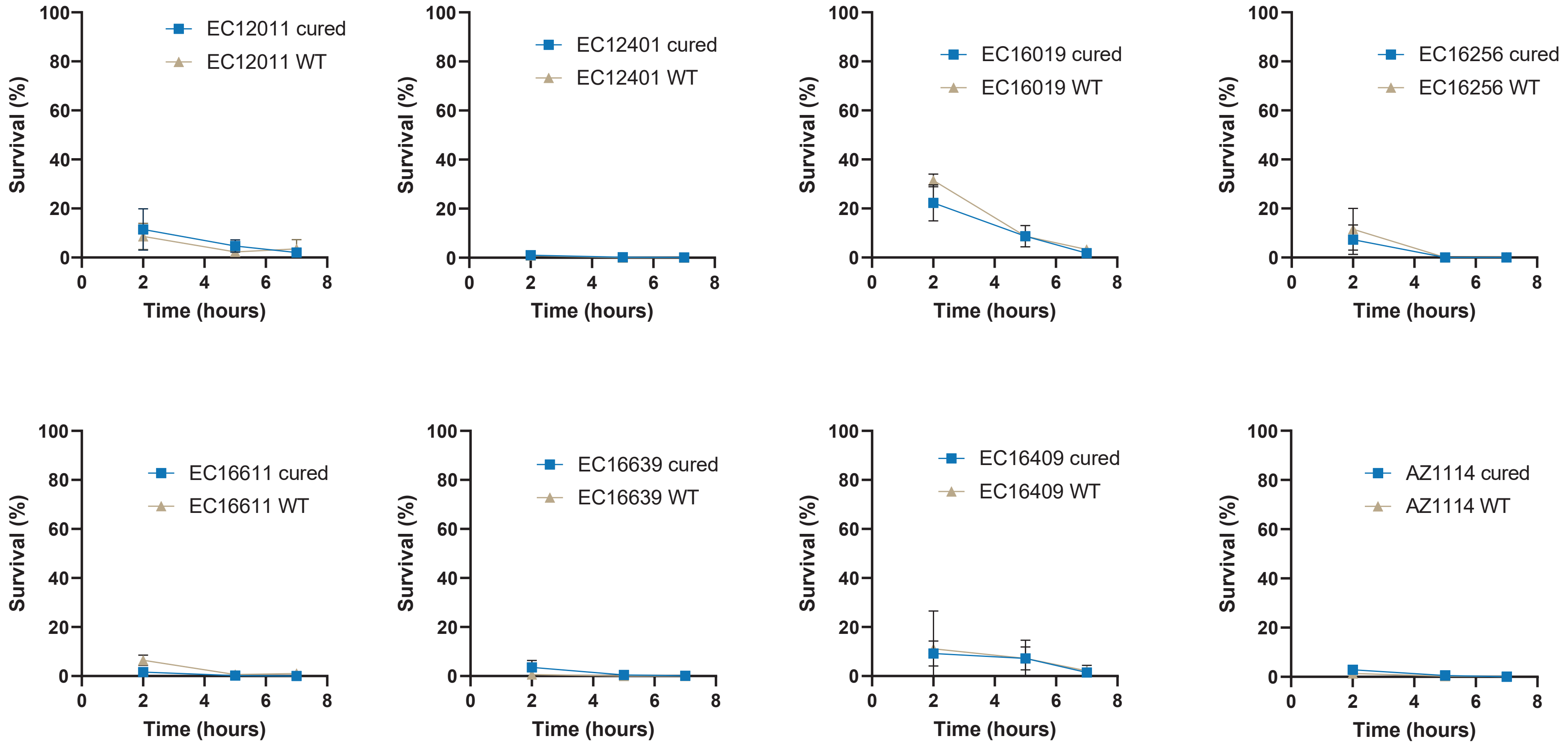

Figure S1. The survival of ExPEC wild-type and IncF plasmid cured mutants in dry environment.

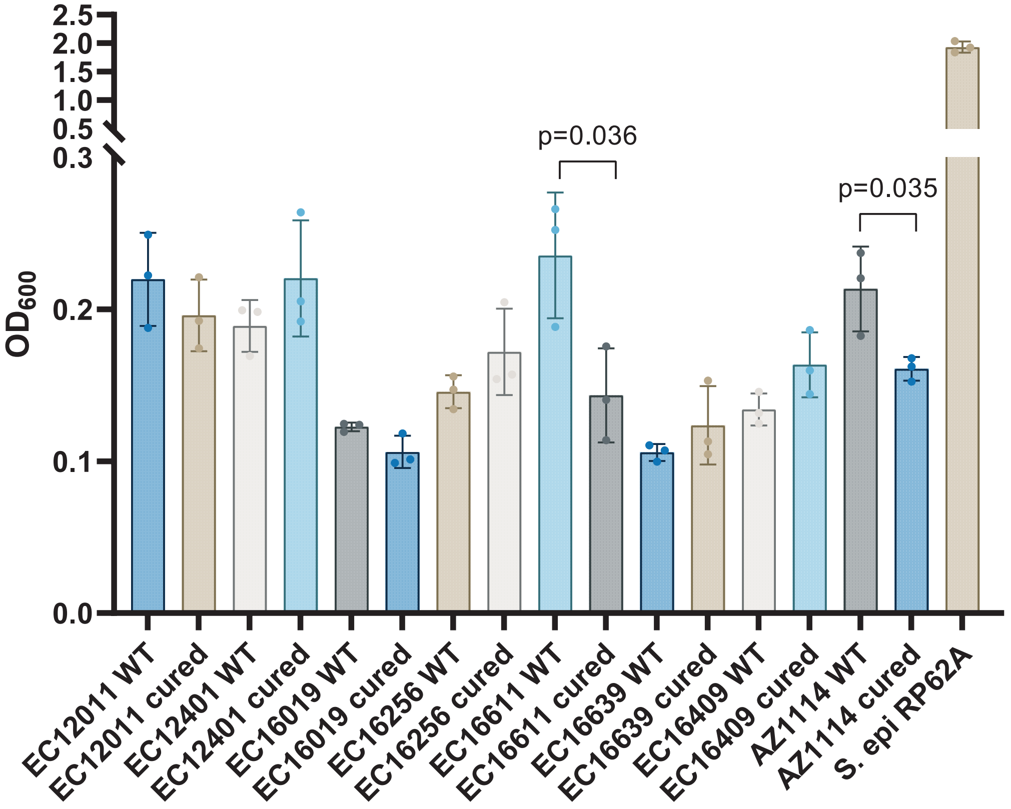

Figure S2. Biofilm production of ExPEC wild-type and IncF plasmid cured mutants
